# Learning curve of EUS-guided gastroenterostomy for the management of malignant gastric outlet obstruction: Data from a bicentric retrospective study

**DOI:** 10.1097/eus.0000000000000221

**Published:** 2026-09-01

**Authors:** Angelica Toppeta, Marie Philippart, Jean-Philippe Ratone, Antoine Assaf, Mariola Marx, Élodie Romailler, Meddy Dalex, Marc Giovannini, Solène Hoibian, Yanis Dahel, Francesco Martini, Sébastien Godat, Fabrice Caillol

**Affiliations:** 1 Endoscopy Unit, Paoli-Calmettes Institute, Marseille, France; 2 Endoscopy Unit, Centre Hospitalier Universitaire Vaudois, Lausanne, Switzerland.

**Keywords:** EUS-guided gastroenterostomy (EUS-GE), learning curve, malignant gastric outlet obstruction, wireless EUS-guided gastroenterostomy simplified technique (WEST), lumen-apposing metal stents (LAMS)

## Abstract

**Background and Objectives:**

EUS-guided gastroenterostomy (EUS-GE) is a promising alternative for managing malignant gastric outlet obstruction (GOO). This study aimed to analyze the learning curve (LC) for EUS-GE.

**Methods:**

This retrospective European bicentric study included 96 patients with malignant GOO who underwent EUS-GE (April 2021 to August 2024) using the wireless EUS-guided gastroenterostomy simplified technique (WEST) or modified WEST. The primary outcome was to define the LC for each center through a composite score based on the clinical success rate, severe adverse events (III–IV per the AGREE classification), and procedural time. Secondary outcomes included the technical success rate, length of hospital stay, and resumption/initiation of chemotherapy.

**Results:**

A total of 218 consecutive patients with GOO were screened across both centers. Twenty-seven patients were excluded because of surgically altered anatomy and/or benign GOO, leaving 191 patients with malignant GOO in the treatment-flow cohort. Among these, 96 patients underwent EUS-GE (Center 1: *n* = 53; Center 2: *n* = 43), whereas 95 patients underwent endoscopic duodenal stenting (Center 1: *n* = 74; Center 2: *n* = 21); no patient underwent surgical gastrojejunostomy during the study period. Ninety-six patients (54% women; mean age 68 years) were included. The technical and clinical success rates were 93% (*n* = 89/96) and 89% (*n* = 80/89), respectively. Severe complications occurred in 6% of patients, with a 1% mortality rate. The main cause of technical failure was misdeployment of the lumen-apposing metal stent, with the distal flange deployed between the stomach and the intestinal wall in 83% of cases (*n* = 5). The LC suggested a stabilization phase after approximately 20 procedures/center. The median length of stay was 8 days (IQR 6–10), and 69% (95% CI 55.6–75.6) of patients resumed or initiated chemotherapy.

**Conclusions:**

EUS-GE is a complex procedure that, at the team level, appears to reach a stable performance phase after approximately 20 procedures, with high clinical success rates and low complication rates.

## INTRODUCTION

Gastric outlet obstruction (GOO) is a debilitating condition caused by mechanical obstruction of the distal stomach or proximal duodenum and frequently results from malignancies such as pancreatic, gastric, and duodenal cancer or cholangiocarcinoma.^[1,2]^ Malignant GOO is a debilitating condition that necessitates prompt intervention to alleviate symptoms, improve nutritional status, and enhance quality of life. Curative options are limited to early-stage disease, leaving palliative interventions as the primary approach for unresectable cases.^[3]^ Historically, surgical gastrojejunostomy (GJ) has been the gold standard for managing GOO, offering high clinical success but carrying risks of significant morbidity and prolonged recovery.^[4,5]^ Endoscopic duodenal stenting has emerged as a less invasive alternative, achieving similar technical success but facing limitations because of tumor ingrowth and stent obstruction.^[6–8]^

EUS-guided gastroenterostomy (EUS-GE) has emerged as a promising alternative, combining the advantages of endoscopic intervention with the durable benefits of surgical GJ.^[9–11]^ By deploying lumen-apposing metal stents (LAMS), EUS-GE creates an anastomosis between the stomach and jejunum, bypassing the tumor site and reducing the risk of stent-related complications.^[12,13]^ The introduction of HOT-LAMS stents and duodenojejunal loop distension has simplified the procedure, making it safer and more efficient.^[14,15]^ Despite promising results, the complexity of the procedure requires a steep learning curve (LC), and the current literature lacks comprehensive evaluations of training dynamics across multiple centers.^[16]^

In this study, the LC of EUS-GE was evaluated using the Wireless EUS-guided gastroenterostomy simplified technique (WEST) and modified WEST, and the clinical success rates, AEs, and procedural time across two centers were assessed to inform future training protocols and standardization efforts.^[14,17–19]^

## MATERIALS AND METHODS

This retrospective, observational, real-world study was conducted at two tertiary endoscopic centers: Center 1 (Institut Paoli-Calmettes, France) and Center 2 (Centre Hospitalier Universitaire Vaudois, Switzerland). Consecutive patients aged 18 years or older with malignant GOO due to unresectable primary gastroduodenal, pancreatobiliary malignancies, or metastasis who underwent EUS-GE from April 2021 to August 2024 were included. In both centers, EUS-GE was considered as a first-line palliative intervention for patients with malignant GOO and an estimated life expectancy of more than three months, in accordance with local multidisciplinary team decisions. All eligible patients underwent computed tomography assessment before the procedure to evaluate suitability for EUS-GE, including the presence, position, and extension of any previously placed duodenal stent. Patients with surgically altered anatomy and/or benign GOO were excluded. Patients with prior duodenal stenting were eligible for EUS-GE if the stent did not extend to or beyond the duodenojejunal angle and did not interfere with safe EUS-guided access to the target small-bowel loop. During the study period, no patient underwent surgical gastrojejunostomy in either center.

All clinical, biological, and paraclinical data were collected in an anonymized manner and recorded in a secured electronic database. Procedural characteristics, such as the technique used (WEST or modified WEST), type of LAMS, and associated biliary drainage, were recorded. The primary outcome was to define the LC, which was assessed using a composite score based on clinical success, severe AEs (III–IV according to the AGREE classification), and procedure time. Clinical success was defined as an increase of at least 1 point in the Gastric Outlet Obstruction Scoring System at 1 month. Technical success was defined as the creation of the gastroenterostomy fistula by the successful placement of a LAMS. Secondary outcomes included technical success, post-procedure LOS, and resumption/initiation of chemotherapy.

All procedures were performed under general anesthesia. A regular upper endoscope was used to introduce a nasojejunal catheter beyond the obstruction, or a transnasal endoscope was passed directly across the obstruction for the modified WEST technique, defined as WEST performed with a transnasal endoscope without the need for a nasojejunal catheter. Sterile water (500–2000 mL) mixed with indigo carmine was injected to distend the jejunal loop. During the procedure, 5 mg of intravenous glucagon or 10 mg of butylscopolamine was injected to reduce intestinal peristalsis. The endoscope was then switched to a linear echoendoscope, which was positioned along the greater curvature of the gastric body to identify the distended small-bowel loop using EUS and fluoroscopy. The LAMS was deployed freehand under EUS and fluoroscopic guidance. The Hot AXIOS 20 × 10 mm LAMS was preferentially selected based on emerging evidence suggesting favorable outcomes compared with smaller-diameter stents.^[16]^ Smaller stent sizes (15 × 10 mm or 10 × 10 mm) were used only when the 20 mm size was considered anatomically inappropriate or unavailable. Some patients underwent additional procedures, such as ERCP or hepaticogastrostomy, for biliary obstruction during the same session.

EUS-GE was implemented in both centers within a team-based interventional EUS setting. Although a primary operator was identified for each case, procedural planning, technical decision-making, and intraprocedural troubleshooting frequently involved other experienced endoscopists. For this reason, the LC analysis was primarily conducted at the center/team level.

Approval was obtained from the institutional review board and the local medical ethics committee. The IRB number assigned to this study was IPC-2023-003 (RETROLAMSDUO).

### Statistical analysis

Continuous variables are reported as mean ± standard deviation or median with interquartile range (IQR), as appropriate, and categorical variables as counts and percentages. LC were assessed at the center/team level by plotting the composite score against the chronological sequence of procedures. Initial exploratory analyses used a smoother (lambda = 0.01), smoothing splines, and a kernel smoother to visualize performance trends over time. To provide a more formal assessment of the transition from the learning phase to the stabilization phase, segmented linear regression analyses were performed on the procedure-by-procedure composite score for each center. Because the exploratory curves suggested stabilization around 20 procedures, a knot at procedure 20 was tested to assess whether the post-threshold slope became negligible. Accordingly, approximately 20 procedures were interpreted as a pragmatic threshold for team-level performance stabilization rather than as an exact universal cut-off. Univariate and multivariate analyses were conducted to assess the impact of patient characteristics (*e.g.*, sex, age, WHO performance status, and BMI) or the type of LAMS on the LC. Statistical analysis was performed using JMP Pro v13.2.1 (SAS Institute Inc., Cary, NC, USA). The composite score was pragmatically developed by the investigators of the two participating centers to reflect the three dimensions considered most relevant to procedural learning: clinical efficacy, safety, and efficiency. Because patient benefit and safety were considered more important than procedural speed alone, clinical success and major AEs were weighted more heavily than procedure time. This score was designed for exploratory assessment of the team-level LC and was not intended as a previously validated instrument.

The composite score was calculated using the following formula:


$$\begin{array}{cc} & Composite\;score=\left(0.4\times Clinical\;Success\right)\\ & +(0.4\left(1-Major\;Complications\right)\\ & -0.2\left(\frac{Procedure\;Time}{Maximum\;Time}\right)). \end{array}$$

## RESULTS

### Patient characteristics

**Figure 1.**
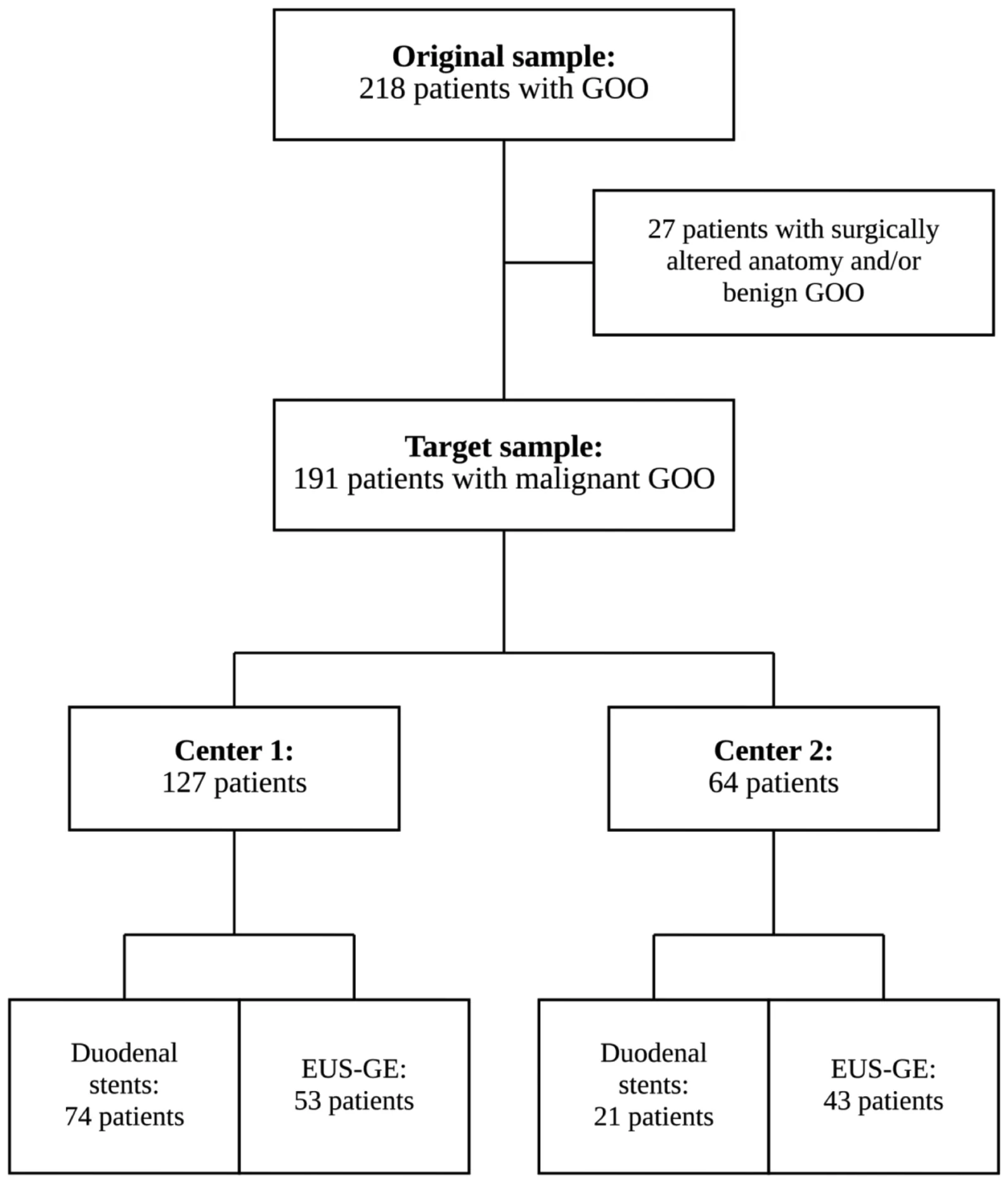
**Flowchart illustrating patient selection and treatment distribution during the study period.** Among 218 consecutive patients with gastric outlet obstruction (GOO), 27 were excluded because of surgically altered anatomy and/or benign GOO, leaving 191 patients with malignant GOO in the treatment-flow cohort. Overall, 96 patients underwent EUS-GE (53 at Center 1 and 43 at Center 2), whereas 95 underwent endoscopic duodenal stenting (74 at Center 1 and 21 at Center 2). No patient underwent surgical gastrojejunostomy during the study period.

**Figure 2.**
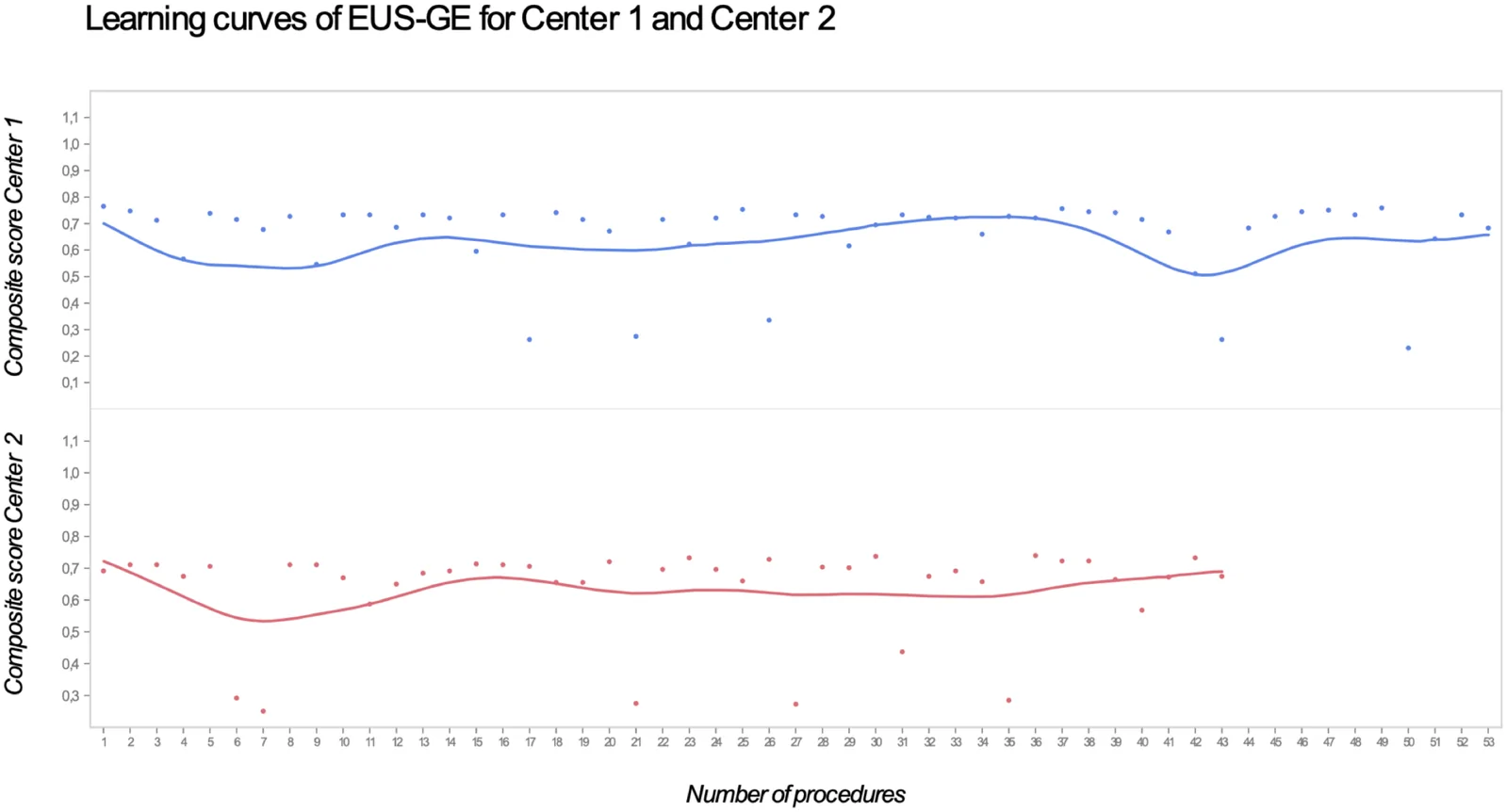
**Composite score plotted against consecutive EUS-GE procedures for both centers** (Center 1, *blue line*; Center 2, *red line*). Each dot represents one procedure. Smoothed curves show an early phase of variability followed by performance stabilization after approximately 20 procedures per center.

During the study period, 218 consecutive patients with GOO were screened across both centers. Twenty-seven patients were excluded because of surgically altered anatomy and/or benign GOO, leaving 191 patients with malignant GOO in the treatment-flow cohort [Figure 1]. Among these, 96 patients underwent EUS-GE and were included in the LC analysis: 53 at Center 1 and 43 at Center 2 [Figure 2]. The remaining 95 patients underwent endoscopic duodenal stenting (74 at Center 1 and 21 at Center 2). No patient underwent surgical gastrojejunostomy in either center during the study period. The final EUS-GE cohort included 44 males and 52 females. The mean age was 68 years (SD 12.8). The most common cause of GOO was pancreatic cancer (66%), followed by metastasis from other cancers (18%), advanced gastric cancer (8%), and cholangiocarcinoma (7%). Eighteen percent of patients had a World Health Organization performance status (WHO PS) ≥2, and 6% had ascites. A total of 46% of patients had prior duodenal stenting [Table 1].

**Table 1:** Baseline characteristics of patients who underwent EUS-guided gastroenterostomy (EUS-GE) at the two participating centers.

	Center 1	Center 2	Total
Sample size	53	43	96
Male (*n*/*N* [%])	27 (51)	17 (40)	44 (46)
Age (yr)			
Mean	67	69	68
SD	10.64	13.67	12.18
Type of cancer (*n*/*N* [%])			
Pancreatic cancer	43 (81)	21 (49)	64 (66)
Cholangiocarcinoma of the extrahepatic biliary duct	2 (4)	5 (12)	7 (7)
Advanced gastric cancer in the antrum or duodenum	3 (4)	5 (12)	8 (8)
Metastasis of other cancers	5 (10)	12 (28)	17 (18)
Metastatic cancer (*n*/*N* [%])	29 (55)	32 (74)	61 (63)
WHO PS ≥2 (*n*/*N* [%])	6 (11)	12 (28)	18 (18)
Duodenal stenting (*n*/*N* [%])	29 (55)	16 (37)	45 (46)
Ascites (*n*/*N* [%])	1 (2)	5 (12)	6 (6)
GOOSS			
Mean	0.71	1.2	0.95
SD	0.66	0.54	0.65

Data are presented as *n*/*N* (%) or mean ± standard deviation, unless otherwise specified.

GOOSS, Gastric Outlet Obstruction Scoring System.

### Procedural characteristics

At Center 1, 53 patients underwent WEST, whereas at Center 2, 35 patients underwent modified WEST, and 8 underwent standard WEST. Hot AXIOS 20 × 10 mm LAMS was used in 88% (*n* = 85/96) of cases. At Center 1, 8% (*n* = 4/53) of patients underwent hepaticogastrostomy, and 6% (*n* = 3/53) underwent ERCP during the same EUS-GE session, whereas at Center 2, 16% (*n* = 7/43) underwent concomitant ERCP [Table 2]. The technical and clinical success rates were 93% (*n* = 89/96) and 89% (*n* = 80/89), respectively [Table 3]. The mean procedure time was 45.74 minutes (SD 2.14).

**Table 2:** Technical characteristics of EUS-guided gastroenterostomy (EUS-GE) procedures at the two participating centers.

Item	Center 1	Center 2	Total
Type of LAMS (*n*/*N* [%])			
Hot Axios—10 × 10 mm	-	1 (2)	1 (1)
Hot Axios—15 × 10 mm	6 (11)	3 (7)	9 (9)
Hot Axios—20 × 10 mm	46 (87)	39 (91)	85 (88)
Hot Spaxus—16 mm × 10 mm	1 (2)	-	1 (1)
EUS-GE technique (*n*/*N* [%])			
Center 1—WEST	53 (100)	8 (19)	61 (64)
Center 2—modified WEST/WEST, *n* (%)	-	35 (81)	35 (36)
Biliary drainage (*n*/*N* [%])			
Hepaticogastrostomy	4 (8)	-	4 (4)
ERCP	3 (6)	7 (16)	10 (10)
Others	-	1 (2)	1 (1)

Data are expressed as *n*/*N* (%).

ERCP, endoscopic retrograde cholangiopancreatography; EUS-GE, EUS-guided gastroenterostomy; LAMS, lumen-apposing metal stent; WEST, wireless EUS-guided gastroenterostomy simplified technique.

**Table 3:** Primary and secondary outcomes of EUS-guided gastroenterostomy (EUS-GE) according to the participating centers.

Item	Center 1	Center 2	Total	*P* value
Clinical success (%, 95% CI)	91.84 (83.89–99.78)	85.00 (73.43–96.57)	88.76 (82.07–95.45)	0.315
Technical success (%, 95% CI)	92.45 (85.10–99.80)	93.02 (85.09–100)	92.71 (87.41–98.00)	0.916
Procedural time (min)				
Mean	43.60	48.37	45.74	0.267
SD	2.89	3.17	2.14	
Major adverse events according to AGREE III–IV classification (%, 95% CI)	5.66 (0–12.09)	7.14 (0–15.27)	6.32 (1.33–11.3)	0.771
Resumption of chemotherapy after EUS-GE (%, 95% CI)	69.77 (55.47–84.07)	67.65 (51.08–84.22)	69 (58.25–79.41)	0.844
Length of hospital stay, median (IQR)	5 (2–7)	12 (7–16)	8 (6–10)	0.006

Data are presented as percentages with 95% confidence intervals, means ± standard deviations, or medians (interquartile ranges, IQRs), as appropriate.

AGREE, Adverse Events in Gastrointestinal Endoscopy; EUS-GE, EUS-guided gastroenterostomy.

### Adverse events

The primary cause of technical failure was the misdeployment of the LAMS, with a distal flange deployed between the stomach and the intestinal wall in 83% of cases (*n* = 5). Three cases were managed endoscopically, while the other two cases required surgery. One case involved the deployment of the distal flange in the peritoneum and the proximal flange in the stomach, resulting in an enterotomy that was managed surgically. The mortality rate was 1%, which was associated with preexisting severe comorbidities. At Center 1, two cases of severe adverse events (AEs) (67%) occurred within the first 20 procedures *versus* 33% in the following procedures. In contrast, at Center 2, all severe complications occurred during the initial procedure (100%), except for one case of mortality, which occurred after 20 procedures [Table 4].

**Table 4:** Overall and center-specific comparison of adverse events (AEs), clinical success, procedural time, and composite score in patients undergoing EUS-guided gastroenterostomy.

Items	≤20 procedures	>20 procedures	*P* value
AEs grade I (*n*, %)	-	1 (2)	1
Center 1	-	1 (3)	1
Center 2	-	-	1
AEs grade II (*n*, %)	1 (3)	1 (2)	1
Center 1	1 (5)	1 (3)	1
Center 2	-	-	1
AEs grade IIIa (*n*, %)	2 (5)	1 (2)	0.549
Center 1	2 (1)	1 (3)	1
Center 2	-	-	0.569
AEs grade IIIb (*n*, %)	3 (8)	-	0.049
Center 1	-	-	1
Center 2	3 (15)	-	0.069
AEs grade V (*n*, %)	-	1 (2)	1
Center 1	-	-	1
Center 2	-	1 (4)	1
Clinical success (*n*, %)	35 (88)	54 (96)	1
Center 1	18 (90)	32 (97)	1
Center 2	17 (85)	22 (96)	1
Procedural time, median (IQR)	36.5 (31.9–51.5)	39.5 (27.4–58.6)	0.861
Center 1, median	33 (30–54)	36 (30–56)	1
Center 2, median	40 (37.5–44)	43 (26.5–59.5)	0.262
Composite score, mean (SD)	0.635 (0.220)	0.648 (0.179)	0.746
Center 1, mean	0.612(0.2777)	0.641 (0.200)	0.665
Center 2, mean	0.658 (0.148)	0.660 (0.148)	0.975

Data are presented as absolute numbers and percentages, or as median and interquartile range (IQR) for continuous variables. The *P* values refer to the comparison between the two centers and to the overall cohort.

### Primary outcome: Learning curve

Both centers exhibited a similar learning pattern, characterized by an early phase of greater variability followed by subsequent stabilization of the composite score. In the original smoothed LCs, this stabilization became visually apparent after approximately 15–20 procedures in both centers. Additional segmented regression analyses supported this interpretation, as the post-20 slope was essentially flat in both centers (Center 2: +0.0017 per procedure; Center 1: −0.00024 per procedure), consistent with a stabilization phase after approximately 20 procedures (Supplementary Table 1, https://links.lww.com/ENUS/A413). The complication rates also tended to decline after the early learning phase, reflecting improved familiarity with the technique.

A total of 11 endoscopists were involved across the two centers (5 in Center 1 [F. Caillol, J.-P. Ratone, M. Giovannini, S. Hoibian, Y. Dahel] and 6 in Center 2 [É. Romailler, M. Marx, M. Robert, S. Godat, S. Oumrani, T. Greuter]). Because operator case volumes were highly heterogeneous, with several endoscopists contributing only a few procedures, individual operator-level LCs were considered insufficiently stable for meaningful interpretation. Teaching was conducted similarly in both centers: one operator was initially trained and then trained a second operator by performing the procedure together. Once two operators were trained, both jointly trained the remaining team members. The analysis, therefore, evaluates the LC of procedure implementation at the center/team level.

### Secondary outcomes

The median LOS was 8 days (IQR: 6–10), and chemotherapy was resumed or initiated in 69% (95% CI 58.25–79.41) of the patients [Table 3]. Patients who achieved clinical success demonstrated a higher likelihood of chemotherapy resumption (*P* = 0.076).

In the univariate and multivariate analyses of the weighted composite score, WHO PS >=2 was the only variable negatively correlated (Pearson -0.298), whereas prior duodenal stenting and the early learning phase were not significantly associated with the score (Supplementary Tables 2 and 3, https://links.lww.com/ENUS/A413).

## DISCUSSION

In this study, for the first time, we evaluated the LC of EUS-GE using WEST and modified WEST across two independent centers, each involving a dedicated endoscopic team. A novel aspect of our analysis was the introduction of an exploratory composite score integrating clinical success, AEs, and procedural time. Unlike previous studies that relied mainly on time efficiency, our composite metric provides a broader evaluation of procedural performance, although it should not be interpreted as a previously validated instrument.

Rather than assessing performance at the individual operator level, we analyzed the LC of an endoscopic team. This reflects the collaborative nature of EUS-GE, which involves close coordination among team members and multidisciplinary input for patient selection and procedural planning. Our data suggest that team-level performance stabilization was observed after approximately 20 cases per center, with a subsequent plateau of the composite score. Our results are consistent with previous reports on the EUS-GE LC. Jovani *et al*. proposed the need for 25 procedures to achieve proficiency and 40 for mastery, whereas Tyberg *et al*. reported improved efficiency after as few as seven cases.^[20,21]^ Recent prospective data on junior endoscopists performing EUS-GE with the WEST technique have also emphasized the importance of evaluating training beyond procedure time alone, incorporating autonomy, technical success, clinical success, fluoroscopy exposure, and AEs.^[22,23]^

During the early learning phase—characterized by variability in technique and increased operator adaptation—AEs occurred more frequently. At Center 1, in particular, two-thirds of all complications were recorded within the first 20 cases. A slight decline in performance was observed at both centers in the later stages of the curve, possibly due to increased case complexity and greater inclusion of frail or high-risk patients as operator confidence increased. These findings suggest that patient-related factors—such as frailty, nutritional status, and advanced oncologic disease—may play a more significant role in complications than procedural inexperience alone does.

Our study extends these findings in two important ways: first, by incorporating a multidimensional exploratory composite score that includes clinical effectiveness and safety, and second, by analyzing multicenter data involving two separate teams in different countries. These contributions enhance the generalizability of the LC and reinforce the validity of EUS-GE as a reproducible and teachable technique.

Our definition of clinical success was based on a sustained increase of at least one point in the Gastric Outlet Obstruction Scoring System after 1 month, which is a more stringent criterion than that used in some prior studies. While others have defined success as the ability to tolerate liquids within days, we evaluated longer-term functional outcomes in a palliative population, including severely cachectic patients. Notably, all patients who achieved technical success were able to resume oral intake the day after the procedure, yielding a 100% short-term clinical success rate, which is consistent with the literature.

This study confirms the feasibility, safety, and effectiveness of EUS-GE as a palliative treatment for malignant GOO, with high technical and clinical success rates and a low incidence of AEs.^[7,11,12,24–26]^ Recent randomized trials comparing EUS-GE with surgical gastroenterostomy have also supported its role as a minimally invasive alternative in malignant GOO.^[27,28]^

In cases of LAMS misdeployment, which is typically manageable using rescue endoscopic techniques, we predominantly used over-the-scope clips or standard clips, in line with current practice. These strategies proved effective in most situations, underscoring the importance of technical versatility during EUS-GE.

A notable proportion of patients (46%) had undergone prior duodenal stenting. Although concern has been raised that duodenal stents may interfere with EUS-GE by complicating access or covering the puncture site, prior duodenal stenting was not considered an exclusion criterion per se in the present study. Rather, patients were considered eligible if the stent did not extend to or beyond the duodenojejunal angle and did not interfere with safe identification of the target small-bowel loop on preprocedural CT assessment. This approach reflects real-world practice in malignant GOO, where EUS-GE may be considered after previous duodenal stenting when anatomical conditions remain favorable.

The variability in the EUS-GE technique highlights the need for procedural standardization and formalized training. Different approaches—such as direct, assisted, WEST, or double-balloon methods—may differ in complexity and thereby impact the LC. As a result, standardized protocols and dedicated training programs are essential to ensure consistent performance and patient safety.

Currently, EUS-GE remains a technically demanding procedure performed in highly specialized centers. There is ongoing discussion in Europe and elsewhere about how best to structure training and evaluate competency in advanced interventional EUS.^[14,17,18,29–32]^ National societies and the European Society of Gastrointestinal Endoscopy are actively working to establish structured curricula and minimum competency thresholds.^[18,29]^

Our analysis also explored secondary outcomes, including hospital length of stay (LOS) and the resumption of chemotherapy. The mean LOS was relatively short (8 days), indicating that the procedure is well tolerated and does not require prolonged postprocedural monitoring. Additionally, despite the advanced disease stage in many patients, chemotherapy was resumed or initiated in a substantial proportion, reflecting the role of the procedure in supporting continued oncologic care. However, further prospective studies are needed to more precisely characterize the impact of EUS-GE on chemotherapy resumption.

This study has several limitations. The retrospective design introduces the potential for selection and reporting bias. All procedures were performed by expert interventional endoscopists in tertiary centers, which may limit generalizability to less experienced operators. The relatively small sample size limited the ability to conduct multivariable analyses for secondary outcomes and to precisely define the threshold for mastery. Mastery (~40 cases) could be estimated only for Center 1, which contributed to the larger cohort. In addition, the composite score used for the LC analysis was investigator-designed and exploratory rather than previously validated. Moreover, our findings apply specifically to the WEST and modified WEST and cannot be extrapolated to other EUS-GE approaches. Finally, the study was limited to malignant GOO, and the results may not apply to benign conditions.

In conclusion, our study provides novel insights into the LC for EUS-GE using an exploratory multidimensional composite score across two centers and endoscopy teams. Performance stabilization was observed after approximately 20 procedures with comparable results across institutions, supporting the generalizability of the technique. As the use of interventional EUS continues to evolve, structured training and standardized protocols are essential for safe and effective implementation. Our findings offer a potential benchmark for independent practice and highlight the value of multicenter research in advancing procedural standardization and training in EUS-GE.

## Supplementary Information

Supplemental digital content is available for this article. Direct URL citations are provided in the HTML and PDF versions of this article on the journal’s Web site (www.eusjournal.com).

## Source of Funding

No financial support was received for this study.

## Ethical Statements

The study was conducted in accordance with the Declaration of Helsinki for biomedical research involving human subjects. Approval was obtained from the institutional review board and the local medical ethics committee. The IRB number assigned to this study was IPC-2023-003 (RETROLAMSDUO). In accordance with institutional policy for retrospective studies using anonymized clinical data, no patient objected to the use of their anonymized data. All data were collected in an anonymized manner and recorded in a secure electronic database.

## Conflicts of Interest

Marc Giovannini has served as a consultant for Pentax and Cook Medical and has received training support from Taewoong Medical. Fabrice Caillol and Jean-Philippe Ratone have received training support from Cook Medical. Marc Giovannini is also one of the Founding Editors-in-Chief of the journal. The article was subjected to the standard procedures of the journal, with a review process independent of the editor and his research group. All other authors declare that they have no conflicts of interest relevant to this manuscript.

## Author contributions

A. Toppeta and J.-P. Ratone wrote the original draft of the manuscript. J.-P. Ratone, F. Caillol, and F. Martini contributed to conceptualization. A. Toppeta, M. Philippart, and A. Assaf contributed to data curation. F. Martini and A. Toppeta performed the formal analysis. A. Toppeta, M. Philippart, J.-P. Ratone, and F. Caillol contributed to the investigation. A. Assaf, F. Martini, J.-P. Ratone, and A. Toppeta contributed to methodology. J.-P. Ratone, M. Giovannini, S. Hoibian, Y. Dahel, M. Marx, É. Romailler, S. Godat, and F. Caillol performed the procedures. All authors contributed to supervision, validation, visualization, data interpretation, critical revision of the manuscript for important intellectual content, approved the final version to be published, and agreed to be accountable for all aspects of the work.

## Data Availability Statement

The data that support the findings of this study are available from the corresponding author upon reasonable request.

## Use of Large Language Models, AI and Machine Learning Tools

During the preparation of this manuscript, the authors used ChatGPT (OpenAI) solely to assist with English grammar and language editing. The authors reviewed and edited all AI-assisted output and take full responsibility for the accuracy, integrity, and final content of the manuscript. Artificial intelligence was not used for data collection, statistical analysis, interpretation of the results, or generation of figures.
